# Meeting the UK Government’s prevention agenda: primary care practitioners can be trained in skills to prevent disease and support self-management

**DOI:** 10.1177/1757913920977030

**Published:** 2021-02-15

**Authors:** W Lawrence, D Watson, H Barker, C Vogel, E Rahman, M Barker

**Affiliations:** Medical Research Council Lifecourse Epidemiology Unit, University of Southampton, Southampton General Hospital, Southampton SO16 6YD, UK; NIHR, Southampton Biomedical Research Centre, University Hospital Southampton NHS Foundation Trust, Southampton, UK; Global Health Research Institute, School of Human Development and Health, Faculty of Medicine, University of Southampton, Southampton, UK; Public Health, Faculty of Medicine, University of Southampton, Southampton, UK; Medical Research Council Lifecourse Epidemiology Unit, University of Southampton, Southampton, UK; NIHR, Southampton Biomedical Research Centre, University Hospital Southampton NHS Foundation Trust, Southampton, UK; Health Education England (Wessex), School of Public Health, Southern House, Otterbourne, Hants, UK; Medical Research Council Lifecourse Epidemiology Unit, University of Southampton, Southampton, UK; NIHR, Southampton Biomedical Research Centre, University Hospital Southampton NHS Foundation Trust, Southampton, UK

## Abstract

**Aims:**

The NHS Long Term Plan has a prevention focus and ambition to support patients to self-manage disease through improving health behaviours. An essential requirement of self-management is behaviour change, but many practitioners have not been trained in skills to support behaviour change. ‘Healthy Conversation Skills’ (HCS) training was developed at the University of Southampton for this purpose. This article reports on a pilot study that aimed to assess the feasibility of primary care practitioners adopting HCS in their routine practice. It describes their experiences and level of competence post-training.

**Methods:**

Health Education England (Wessex) commissioned HCS training for 18 primary care practitioners. Fifteen of these practitioners were subsequently observed in their consultations at one or two time points; face-to-face semi-structured, reflective feedback interviews were conducted immediately following the observations. Practitioners’ HCS competence was assessed from the observations and interviews using a previously developed and published coding rubric. The interview data were analysed thematically to understand practitioners’ experiences of using the new skills.

**Results:**

Practitioners demonstrated competence in embedding the skills into their routine practice following HCS training. They reflected on how patients liked being asked questions, the usefulness of setting SMARTER (Specific, Measured, Action-oriented, Realistic, Timed, Evaluated and Reviewed) goals and the power of listening. They could also identify facilitators of skill use and ways to overcome challenges such as patients with competing priorities and organisational constraints. They found the skills valuable as a way of empowering patients to make changes to manage their own health.

**Conclusions:**

HCS are acceptable to primary care practitioners, can be readily adopted into their routine consultations and are a helpful strategy for supporting patients to make changes. HCS training has the potential to be a sustainable, scalable and effective way of contributing to the prevention agenda by supporting disease self-management, and hence of addressing today’s epidemic of lifestyle-related conditions.

## Introduction

The recently announced NHS Long Term Plan describes ambitions to give patients more control over their health and treatment, and prevent non-communicable diseases, such as cardiovascular and chronic respiratory diseases, most cancers and diabetes, through improving health behaviours.^1^ Disappointingly, it offers no detailed plan for how these ambitions will be realised and appears to place responsibility for this on individual NHS trusts and local authorities. Previous NHS plans were more specific in that they suggested an approach to disease prevention that involved frontline staff in ‘Making Every Contact Count’ (MECC), but it was left to individual trusts and local authorities to decide how best to equip their staff to do this.^2,3^ Taking up this challenge, all regions of England have now established mechanisms for MECC, facilitating health improvement and disease prevention through the development of the wider healthcare workforce. MECC as a principle takes advantage of the fact that everyday more than a million people in the UK have contact with healthcare and social care practitioners. These contacts represent opportunities to support members of the public to adopt healthier behaviours and in so doing ‘make every contact count’.^4^ The MECC agenda provides one way of delivering the UK Government’s growing disease prevention agenda and recognises the opportunity that the workforce has to influence health behaviour and in so doing prevent disease.

Over the last 5 years, National Health Service (NHS) commissioners have included a commitment to MECC principles in standard NHS contracts and linked it directly to CQUINs (Commissioning for QUality and INnovation) relating to self-management.^5^ More recently, the role of the primary care workforce in supporting secondary prevention has been given focus by the announcement of a UK Government emphasis on prevention with the NHS Long Term Plan.^1^

Developing Integrated Care Systems and Primary Care Networks, which are expected to be key in delivering many of the commitments in the Long Term Plan, will place new demands on the workforce for skills in disease prevention.^6,7^ Training the primary care workforce in skills to support healthier behaviours and disease self-management offers one way of meeting these demands.

Recent evaluations of methods used to deliver MECC have concluded that it is acceptable to, and valued by, a range of practitioners and that training in behaviour change skills can benefit patients.^8,9^ Concerns have been raised, however, about the feasibility of implementing the MECC agenda in organisations with varying cultures and structures. Organisations that view themselves as responsible for prevention rather than treatment and have strong relationships between departments demonstrate greater implementation.^9^ Others with organisational and financial constraints have needed strong leadership and commitment to the training of staff and highly engaging and effective training to justify release of staff time.^10,11^ Outside the MECC context, many frontline practitioners do not routinely receive training in skills to support behaviour change. Many report lacking confidence in initiating conversations about diet and exercise, for example.^12,13^

Health Education England is responsible for the training and development of the healthcare workforce. In 2013, the Wessex region proposed using Healthy Conversation Skills (HCS) as the mechanism for delivering MECC. In a range of evaluations, HCS-trained health and social care practitioners had shown improved confidence and competence in supporting behaviour changes, demonstrating continued use of the skills up to one year post-training.^14–16^ HCS training adopts an empowering, person-centred approach to changing behaviour. It equips frontline practitioners with skills to maximise the benefit from conversations with patients that support them to find their own solutions and identify first steps to change.^17^ HCS are practical, simple to learn and can be used opportunistically by practitioners working in any setting, with any population, in any time frame. Box 1 outlines the training philosophy and programme; further details have been published elsewhere.^17^

The aim of this study was to evaluate the feasibility of training primary care practitioners from Wessex in HCS and to assess how they embed the skills into routine practice. Such practitioners play a key role in preventing disease in local communities and in delivering the UK Government’s commitment to ‘putting prevention at the heart of our nation’s health’^18^ (p. 4). Staff working within primary care settings have opportunities to discuss a wide range of health behaviours, including diet, physical activity, smoking, alcohol consumption and medication management.

This article addresses the following research questions:

1. How well do HCS trainees demonstrate sustained use of the skills post-training?
2. What are primary care practitioners’ experiences of HCS training?
3. How do primary care practitioners implement the skills in routine practice?

## Methods

### Setting and participants

In 2013, Health Education Wessex contacted general practitioner (GP) practices in Hampshire and Buckinghamshire inviting them to pilot the HCS training. An information sheet and booking form were emailed to all GP practice managers via the local clinical commissioning group. GP practices that expressed willingness to take part were asked to release two of their practice staff to attend training. These practitioners were offered HCS training as part of their continuing professional development (CPD) and were sent the training information sheet. Reasons for non-participation included time constraints, too far to travel and insufficient notice. Participants were therefore self-selected. The training was conducted by two HCS trainers with support from another team member; all are experienced in group work and behaviour change. The pilot study was part of the feasibility stage for Health Education Wessex, with the ambition to upscale the training to practitioners across the region.

### Ethics

As HCS training and follow-up were considered service provision and workforce development, the study met the criteria for service improvement and did not, therefore, require approval from the local research ethics committee. However, universal ethical principles were followed throughout the study, and participants were asked for consent to participate at each stage.

### Procedures, materials and data analysis

This longitudinal pilot study is based on data derived from pre- and post-training evaluations, observations and reflective feedback interviews; the latter two activities were undertaken at two time points: 1–2 months and 11–13 months post-training.

#### Pre- and post-training evaluations

All trainees completed an evaluation sheet at the start of Session 1 and end of Session 2 to measure change in use of Open Discovery Questions (ODQs, beginning with How or What) by responding to four statements about diet, exercise, alcohol and smoking. Responses were coded using a previously developed coding matrix.^14^ Changes in confidence, importance and usefulness in relation to supporting change were also measured at these two times on a published 10-point Likert-type scale.^15^

### Observations

Practitioners were observed during one of their routine clinics at their general practice to assess how they used HCS. Observers recorded the use of three of the four HCS (ODQs, Listening and SMARTER (Specific, Measured, Action-oriented, Realistic, Timed, Evaluated and Reviewed) goal-setting) using a standard proforma. Immediately following each observation, observers recorded a competency score for the participant for use of the three HCS using a published competency-rating rubric.^14,15^ Each HCS was scored from 0 to 4, where 4 demonstrated the highest competency. Given the small sample size, formal statistical tests were considered inappropriate and only descriptive statistics were calculated.

#### Reflective interviews

Once all the observations had been completed, the observer conducted a face-to-face reflective feedback interview with the practitioner using a semi-structured discussion guide. Consent was sought from each participant to audio-record the interview. Immediately following the interview, the researcher recorded a competency score for the final HCS (Reflection) based on the interview data, using the coding rubric described above.

The audio-recordings were transcribed verbatim. Using deductive coding and a constant comparative approach,^19^ themes and sub-themes were generated and used to produce a coding framework. Stages in the analysis included (1) review of the transcripts; (2) development of a coding framework to represent emergent themes, illuminated with verbatim quotations; (3) thematic coding of the transcripts using the coding framework; and (4) repeating stages 1—3 until the coding framework was fit-for-purpose. The research team met to discuss any minor disagreements, and a proportion of the transcripts was double-coded to inform the final coding framework.

## Results

Data were collected from clinics covering a range of conditions including sexual health, chronic obstructive pulmonary disease (COPD), cardiac health, overweight, smoking, diabetes, and NHS health checks. Eighteen participants completed the HCS training, ranging in age from 20 to 60 years, with a mean of 10 years’ relevant work experience. Three participants had previously received brief motivational interviewing training. Each reflective interview lasted 15–50 min in length. Data included in the analysis are derived from 15 participants who attended both HCS training sessions and were observed post-training during at least one clinic session and one follow-up reflective interview.

### How well do HCS trainees demonstrate sustained use of the skills post-training?

Table 1 presents measures of HCS competence for individual trainees assessed using three methods: (1) pre- and post-training evaluation sheets; (2) observations of one or two clinic sessions following training; and (3) during reflective interviews carried out after each clinic session.

**Table 1.** Participants’ individual Healthy Conversation Skills (HCS) confidence, importance, usefulness and competence scores (*n* = 15 at FU1; *n* = 9 at FU2)

ID no.	Job role	No. of Open Discovery Question (ODQ) responses		Confidence		Importance		Usefulness		Observation: HCS competence						Reflective interview	
										ODQs		Listening		SMARTER		Reflection	
		Pre-training	Post-training	Pre-training	Post-training	Pre-training	Post-training	Pre-training	Post-training	FU1	FU2	FU1	FU2	FU1	FU2	FU1	FU2
002	Practice Nurse Manager	1	4	7	7	10	10	7	10	4	2	2	2	2	2	4	3
003	Healthcare Assistant	1	4	10	10	10	10	8	10	4	3	2	2	2	1	4	3
004	Healthcare Assistant	0	4	4	10	6	10	8	10	2	3	2	2	1	1	4	4
005	Practice Nurse	3	4	7	10	10	10	8	10	4	4	4	4	4	4	4	4
006	Practice Nurse	0	4	7	9	10	5	10	10	3	4	2	2	2	3	4	4
008	Triage Nurse	0	4	3	8	10	10	10	10	3	4	2	3	1	3	4	4
020	Practice Nurse	1	4	8	9	10	10	8	10	4	4	4	3	4	3	4	4
022	Practice Nurse	0	4	8	7	10	10	8	10	4	4	4	3	2	2	4	4
025	Healthcare Assistant	0	3	7	7.5	10	10	9	10	2	2	2	2	1	2	4	2
009	Practice Nurse	2	4	7	7	9	9	8	8	4	−	2	−	2	−	4	−
011	Dietitian	0	4	9	9	10	9	9	9	4	−	3.5	−	2	−	4	−
021	Practice Nurse	1	4	6	9	8	10	7	10	4	−	4	−	2	−	4	−
024	Occupational Health Nurse	3	−	7	6	9	9	7	9	4	−	4	−	1	−	4	−
026	Sexual Health Nurse	0	4	7	8	10	10	7	8	4	−	1	−	2	−	4	−
027	Occupational Health Nurse	0	4	6	8	8	10	6	8	3	−	2	-	1	−	4	−
Total ODQs		12 (*n* = 15)	55 (*n* = 14)														
Median				7	8	10	10	8	10	4	4	2	2	2	2	4	4

FU1: follow-up 1–2 months post-training; FU2: follow-up 11–13 months post-training. SMARTER: Specific, Measured, Action-oriented, Realistic, Timed, Evaluated and Reviewed.

From pre- to post-training, participants’ use of ODQs (rising from 12 to 55 ODQs), measures of confidence (median score rising from 7 (range: 3–10) to 8 (range: 6–10, out of 10)) and usefulness (median score rising from 8 (range: 6–10) to 10 (range: 8–10, out of 10)) in using the skills to support change, all increased post-training.

Clinic sessions observed at follow-up lasted 1–3 h. The levels of competency for asking ODQs and Reflection were high among participants at both follow-ups (median scores of 4 out of 4 for each at both follow-ups), while Listening (median scores of 2 out of 4 at both follow-ups) and Goal-Setting (median scores of 2 out of 4 at both follow-ups) were moderate.

Table 1 shows the scores for individual trainees as they progressed through training and into using the skills in practice. Their scores illustrate the variation in skills at baseline and in competencies after training and over time as they are implemented. Overall, the data show that competencies stayed relatively stable at most time points for almost everyone.

### What are primary care practitioners’ experiences of HCS training?

In the reflective interviews, practitioners were generally positive as to how the training had affected their skill development. They appreciated the training structure, activities, interactive/participatory style of delivery (no PowerPoint slides), resources and the opportunity to learn with other health professionals:

*I liked, although it was horrible, listening to it back. It was quite nice to do the recording and see where you go wrong and how to change that, sort of reflecting back on yourself.* (ID004_FU1)

*I liked meeting people from other surgeries and having to work with someone you wouldn’t necessarily have ever met before* … (ID004_FU1)

The practitioners considered the training valuable in providing new learning. It was stimulating and useful, encouraging them to reflect on key principles of their practice and how effective the skills were in enabling them to empower patients to identify their own solutions and goals. The value afforded to the skills learnt in the training, in turn led to the perception that the time allocated to post-training, follow-up was important and useful if they were to effectively support their patients to make health changes:

*It’s really cool to have that feedback from you as well, because you’re kind of seeing what I’m not seeing … so that’s pretty cool to have that kind of follow-up.* (ID005_FU2)

### How do primary care practitioners implement the skills in routine practice?

Thematic analysis identified three themes that answer this question. These were (1) how practitioners implemented HCS; (2) what challenges to implementation were experienced; and (3) recognising the impact of having healthy conversations.

#### How practitioners implemented HCS

Practitioners described ways in which they were using HCS and successes they had experienced. They talked about their use of goal-setting and SMARTER techniques in supporting patients to make plans for change:

*I felt it went pretty well and patients have been able to give me some feedback on how their goals went.* (ID005_FU1)

*I will use that SMARTER plan that you gave us … you know Specific and Timed and everything, and try and build a plan with them, especially about weight loss.* (ID004_FU1)

Practitioners reflected on how they were asking ODQs to empower people to find their own solutions and on the effect this had of opening up conversations with patients in a way that revealed more about the patient’s life and context in which they were managing their health condition:

*We talked about his life, what he was doing … what his employment was, what he wanted to do and things …* (ID002_FU2)

*I’m surprised how open people are really … they even say thank you for asking the question.* (ID008_FU2)

There were discussions about listening rather than telling or giving information and reflecting on having healthy conversations. Some spoke of their increased awareness of behaviour change techniques and the impact of HCS overall on their practice:

*I’m doing less talking which is quite good, and trying to do more listening.* (ID020_FU1)

*I think probably I was quite reflective, and I think that I handed the responsibility back to him.* (ID008_FU2)

Practitioners reported, and were observed to be, spending a significant amount of time using and reflecting on all four HCS. The reflective feedback interviews offered practitioners an opportunity to plan further development of their skills, particularly when they recognised occasional use of their previous communication style:

*… maybe sometimes I don’t always listen as much as I should do … I tend to like stepping in for the patient.* (ID006_FU1)

Practitioners reflected on the challenges to using HCS in practice. With guidance and support from the interviewer, practitioners identified ways to practise the skills in order to increase their use:

*Because I do a lot with COPD patients. For me it’s [about] smoking cessation, and I just want to continue practising what I am doing [with the skills] actually.* (ID002_FU2)

*Even if it was only a more open conversation, you know … perhaps might be the aim.* (ID008_FU2)

Practitioners’ commitment to embed these skills in their work is evidenced by their willingness to plan ways in which they could improve.

#### What challenges to implementation were experienced?

Practitioners discussed challenges created by the nature of the job they do, specifically patient attributes and systemic constraints in their workplace and the wider NHS context.

A patient’s medical condition was seen to sometimes preclude the practitioner from having a healthy conversation:

*She wasn’t wearing her hearing aids … if she wasn’t hard of hearing, she would have been a hard patient because she was … not forthcoming.* (ID003_FU2)

Sometimes the habitual nature of a patient’s behaviour was perceived to get in the way:

*It’s her choice … She’s drawing the line and saying to me ‘no, I’m not going to alter that, that’s not negotiable’. I’m not sure that there’s anything else that I can do.* (ID008_FU1)

A patient’s circumstances or general attitude towards health or changing behaviour emerged as a barrier to the practitioner being able to support them effectively:

*I think she’s hesitant because she’s in a bad place. She’s stressed, she’s not coping, and I’m trying to help her, but her finance . . .* (ID003_FU1)

Practitioners responded to patients’ reactions within the clinic when deciding how far they could pursue a healthy conversation with them. How the patient was feeling physically or mentally, and any habitual behaviour affected practitioners’ use of HCS, particularly when patients were considered resistant.

Practitioners were also impeded by the nature of their job role or the wider work context, including mandatory reporting requirements. These included time pressures, the culture of information-giving and the expectation to signpost patients onto other professionals or organisations:

*I get 10 minutes per patient, unless it’s for hearing or ECG, in which I get 20 minutes. But basically it’s in and out … you need to be good at time-keeping.* (ID004_FU2)

*I think nurses are great at kinda telling patients what to do.* (ID006_FU1)

*By the same token, I still have to follow what the NHS wants.* (ID003_FU2)

Expectations remain on professionals to inform patients via leaflet provision or service signposting, sometimes hindering opportunities for having healthy conversations.

#### Impact of having healthy conversations

There was much discussion on how using HCS in consultations had a positive impact on both the patient and the practitioner. HCS were commonly used to build rapport with patients, leading them to be more likely to open up. Engagement in the conversations initiated by the practitioner subsequently led to patients achieving their behaviour change goals:

*It encourages us to be more open with the patient asking them open-ended questions, asking the patients to talk. … I think it’s also building up that relationship with them.* (ID00_FU2)

*This week I saw him, and he came up to me and he put his arm around me and said ‘thank you so much. I haven’t smoked since that morning’.* (ID002_FU1)

Using HCS in both their professional and personal life increased practitioners’ confidence to make changes to their own behaviours and to support patients’ or friends’ health changes:

*Speaking to patients, I’ve got more confident and as I’m progressing in my job role it’s definitely helping me more.* (ID004_FU2)

*My best friend is getting married in 2015 and she wants to be at least two dress sizes smaller, so that’s her goal. I would like to be a dress size smaller, so we’re starting early, so that we can do it properly and slowly, with exercise and everything.* (ID004_FU2)

Recognition of the benefits of using HCS in their practice also prompted many to identify ways that HCS could or should be more widely available to their colleagues:

*I shared the literature you gave us … several of my colleagues have taken it and I know they would be really keen to do it.* (ID002_FU1)

## Discussion

The aims of this study were to explore the experiences and competency levels of primary care practitioners using HCS in their practice following HCS training and to evaluate whether it is feasible for other practitioners to be trained and to embed these skills into routine practice. Findings are discussed below as they answer each study question.

### How well do HCS trainees demonstrate sustained use of the skills post-training?

As seen in previous evaluation of HCS training, primary care practitioners were better able to use some HCS than others.^14^ Observations of patient interactions in clinic at two time points post-training indicated that practitioners were skilled at asking ODQs and reflecting on their practice. But while they had incorporated listening and supporting their patients to set SMARTER goals to some extent, they found these skills more challenging to implement. Reflective interviews suggested that some practitioners felt a healthy conversation was too time-consuming to carry out in addition to completing the required routine tasks. However, all practitioners demonstrated at least moderate competency for each HCS, which indicates that they were able to embed the new skills to some extent into their practice. Thus, HCS can be considered to be acceptable, practical and sustainable up to one year post-training in a primary care setting.

### What are primary care practitioners’ experiences of HCS training?

Practitioners valued the training and described how it enhanced their motivation to use HCS. They felt supported to reflect on how they were using them in their practice and enjoyed the follow-up observations and reflective feedback interviews because they reinforced the value of the skills. They also remarked on the benefits they gained from training together with practitioners from other GP practices. This pilot study suggests that healthcare practitioners with different levels of experience acquire HCS readily and value the way the training encourages them to share good practice.

### How do primary care practitioners implement the skills in routine practice?

All practitioners who were interviewed reflected on how they were using HCS with their patients and the resultant positive impact. Some practitioners still found it difficult not to revert to telling their patients what to do although they understood that this might not be the most effective strategy. The follow-up interview gave practitioners the opportunity to recognise and reflect on this, to make plans to practise and build confidence in using HCS. There were certain types of patients, particularly those with significant competing priorities in life, with whom practitioners found it more challenging to use the skills. Short appointment times were cited as an additional challenge; however, if the NHS Long Term Plan to make every part of the country an integrated care system by 2021 is actioned, it will likely facilitate time for having healthy conversations to support MECC.^20^ Previous research identified systemic barriers to implementing training in skills to support behaviour change. For example, public health practitioners valued the MECC training they were given but were prevented from making best use of their skills because they lacked management support and resources.^10^ Others suggest that ‘the (MECC) service is only as effective as the system in which it operates’^9^ (p. 660).

Practitioners noted that the training had impacted their patients, themselves, and their family, friends and colleagues. They spoke about enjoying their new-found rapport with chronically ill patients, who were more likely to open up about their physical and emotional wellbeing and make plans to manage their health. They saw some improved health outcomes at their follow-up appointments. Barrecheguren and Bourbeau^21^ describe the use of self-management programmes by those with COPD, highlighting the key role that plans and goals play in the success of self-management. Others have reported a 70% increase in uptake of smoking cessation services among patients who had support from practitioners using a MECC approach.^9^ These findings suggest that training in accessible behaviour change skills could have benefits for patients and the broader public if widely implemented. Practitioners spoke of their increased confidence in using HCS with friends and family, suggesting that HCS training could have an impact on the quality of life of practitioners and their social network. Though only hinted at in these data, this presents the intriguing possibility that adopting these skills might have a generalised impact on health and wellbeing.

## Implications for Policy and Practice

### How does HCS represent a means of delivering on the NHS Long Term Plan?

These study findings have implications for disease prevention and exacerbation through encouraging patient self-management in primary care. Using HCS does not require extensive training or familiarisation with complex concepts. The authors would also propose that as practitioners’ confidence and competence in using the skills increase, they should be able to adopt this approach within the time they have available. Practitioners can use the skills in any primary care context and do so over the long term. These attributes suggest that it is likely to be cost-effective. Nelson et al.^9^ said of MECC training, ‘its strength is its simplicity’, observing that this simplicity encouraged organisation and stakeholder commitment.

In order to realise the ambition of the NHS Long Term Plan, health and social care practitioners of all types need skills to support health behaviour change. There is a substantial body of evidence to suggest that practitioners across the NHS currently feel they lack confidence and skills to have effective conversations with patients and clients about weight, diet and other health behaviours.^12,13,22^ The study reported in this article suggests that HCS training could provide primary care practitioners with accessible and easy-to-use skills to empower patients to maintain health and prevent disease exacerbations.

The NHS Long Term Plan assigns responsibility for delivering its preventive agenda to individual NHS trusts and local authorities. While this is, on the one hand, a heavy responsibility, it also presents an opportunity to adopt a trust-wide or local authority-wide approach to prevention, on the other. The colleagues of practitioners trained in the study reported here, expressed a desire to also have training in HCS. There was clearly a willingness in these GP practices to acquire the skills needed to have conversations to support health. Training of all health and social care practitioners across a trust or local authority would create an environment where patients and the public were offered opportunities to reflect on their health needs, make plans and set goals in every meeting with every type of practitioner – truly ‘Making Every Contact Count’.^3^

### Strengths and limitations

This study collected data from a small, self-selected convenience sample of GP practices in Hampshire and Buckinghamshire, thus limiting the generalisability of the findings. As a feasibility study, however, it allowed an exploration of the acceptability and usefulness of the skills in routine primary care practice. It may be that these practitioners are no different in any systematic way to other practitioners in similar roles, but further research is needed to confirm this. Evidence of generalisability comes from consistency with previous data on use of HCS training with larger numbers of health and social care practitioners.^14,15^

Practitioners may have behaved differently while being observed, thus producing observer bias. Equally, the training team interviewing practitioners may have, by nature of their role as a trainer, caused them to overstate evidence of HCS use. However, from the outset, the research team was aware of areas for potential bias and used a wide range of methods in an attempt to address these, including using a variety of evaluation tools, assessing practitioners at multiple time points and collecting several sources of data from each trainee. While evaluation tools were not validated against other instruments, they have been extensively piloted by the research team and found to be fit-for-purpose.^14,15^

## Conclusion

This article indicates that HCS training is a good investment, valued by staff who use the skills in the long term. We propose that HCS are readily adopted by practitioners because the training design and skills match practitioners’ own perceptions of their needs. Having ‘healthy conversations’ does not add to their burden.

Although there has been a delay in publishing these data from the 2013 pilot programme, the findings seem as relevant today as they were then. There are important reasons for publishing now the development work that underpins current HCS training and evaluation activities.

First, a 2019 sandpit activity convened by Health Education England to develop a plan for assessing the national impact of MECC (and attended by two of the authors) highlighted the limited evaluation of very brief interventions such as MECC. This lack of evidence indicates a need for studies such as the one we report in this article in order to inform practitioners, service commissioners and policy makers as to the value of a MECC approach.

Second, this pilot study informed the development and large-scale roll-out of the Wessex MECC programme across three of the five English regions as the method of training health and social care staff to make every contact count.^3^ Since 2014, over 5000 staff have been trained in HCS using a Train-the-Trainer model, and the training is now accredited by the Royal Society for Public Health. A large-scale evaluation is planned, but the pilot data reported in this article are important for establishing the early credibility and usefulness of HCS and to explain why the roll-out was commissioned.

Finally, HCS have proved to be particularly important to practitioners working during the COVID-19 pandemic. Aspects of the face-to-face training described in this article have been adapted for delivery online to support those working with the most vulnerable at this time. To date, the 90-min online ‘Supportive Conversations’ training has been delivered to nearly 300 frontline workers from across England.

In the same way that HCS training is valued by trainees because it is designed to fill a skills gap that they identify for themselves, HCS have been taken up at an organisational level because they meet the requirement in many NHS contracts to deliver on the MECC agenda. There is potential for this training to be fundamental to delivering the preventive ambition of the current NHS Long Term Plan, as well as supporting the UK’s COVID-19 response and recovery, demonstrating the versatility and broad applicability of HCS.

## Appendix 1

### Healthy Conversation Skills training outline

Communication is enhanced through practitioners developing the skill of asking open-ended, or open discovery, questions – those that begin with ‘how’ and ‘what’. Such healthy conversations allow a patient or client to explore an issue, identify barriers and generate solutions that can be reviewed with the practitioner at their next encounter. Experiential training aims to increase self-efficacy and sense of control of both practitioners and their patients and clients.

The four core skills are the following:

1. To use Open Discovery Questions (those that specifically support exploring of issues, barriers and priorities; problem-solving; and goal-setting).
2. To reflect on practice.
3. To listen rather than provide information.
4. To support goal-setting through SMARTER (Specific, Measurable, Action-oriented, Realistic, Timed, Evaluated, Reviewed) planning.

Healthy Conversation Skills training typically consists of two 3- to 4-h group sessions over 1 week to allow time for practising and reflecting on skills. Training is delivered by one or two facilitators experienced in group work and behaviour change to a group of around 6–16 trainees. This is followed by a period of on-going support, which may include a phone call or visit from one of the trainers to find out how skills are being implemented in practice. The phone call/visit allows trainees to reflect on the training, how they have implemented their new skills, any barriers to their implementation and plans for continued or increased use, including embedding self-reflection and peer reflection as part of normal practice. All follow-up activities are also opportunities to collect evaluation data to assess the effectiveness of the training. Undertaking these activities from 1 month post-training is based on an assumption that staff would have opportunities to practise their new skills, and if they were finding this challenging, it would be a good time to reflect on this and make plans for progress. Further follow-ups can be undertaken at later stages to assess long-term use of the skills in practice.
